# *In Vitro* Anti-Inflammatory, Anticancer (MCF-7, 3T3, and HeLa Cell Lines), and Brine Shrimp Lethality Assay and FTIR Analysis of the Extract and Fractions of the Whole Plant of *Heliotropium europaeum*

**DOI:** 10.1155/2020/5056897

**Published:** 2020-02-01

**Authors:** Jahangir Khan Achakzai, Muhammad Anwar Panezai, Basira Akhtar, Shahabuddin Kakar, Ali Akbar, Abdul Manan Kakar, Javed Khan, Nazima Yousaf Khan, Ghulam Mustafa Khan, Muhammad Imran, Marina Panezai, Nisar Ahmed Shahwani, Tehmina Achakzai

**Affiliations:** ^1^Institute of Biochemistry, University of Balochistan, Quetta 87300, Pakistan; ^2^Department of Botany, University of Balochistan, Quetta 87300, Pakistan; ^3^Department of Zoology, University of Balochistan, Quetta 87300, Pakistan; ^4^Department of Microbiology, University of Balochistan, Quetta 87300, Pakistan; ^5^Department of Microbiology, Quaid-i-Azam University, Islamabad 45320, Pakistan; ^6^Department of Chemistry, University of Balochistan, Quetta 87300, Pakistan; ^7^Faculty of Pharmacy, University of Balochistan, Quetta 87300, Pakistan

## Abstract

In this study, anti-inflammatory, anticancer, brine shrimp lethality, and FTIR studies were evaluated. The oxidative burst assay using the chemiluminescence technique, MTT assay, brine shrimp lethality assay, and FTIR analysis were the methods used for the evaluation of anti-inflammatory, anticancer, brine shrimp lethality, and FTIR studies, respectively. The whole-plant butanol fraction of *Heliotropium europaeum* (WBFHE) showed anti-inflammatory activity on ROS having IC_50_14.7 ± 2.5 while the extract and other fractions of the whole plant of *Heliotropium europaeum* exhibited no anti-inflammatory activity. None of the extract and fractions of the whole plant of *Heliotropium europaeum* exhibited anticancer (MCF-7, 3T3, and HeLa cell lines) activities. The whole-plant aqueous fraction of *Heliotropium europaeum* (WAFHE) and whole-plant butanol fraction of *Heliotropium europaeum* (WBFHE) showed lethality at high concentration while at low concentration, no toxicity was shown. The whole-plant methanolic extract of *Heliotropium europaeum* (WMEHE) and whole-plant n-hexane fraction of *Heliotropium europaeum* (WHFHE) exhibited no toxicity. FTIR interpretation showed the functional groups for the aromatic compounds, phenols, carboxylic acids, esters, alkanes, alkenes, alcohols, alkyl halides, sulfate esters, phosphines, silanes, nitriles, thiols, amines, phosphoric acids, and nitro compounds.

## 1. Introduction

With the beginning of folk medicine, the usage of medicinal plants and their incorporation into allopathic and traditional medicine have a long history [1]. Medicinal plants have effective pharmacological activities due to the presence of secondary metabolites such as alkaloids, saponins, tannins, terpenoids, flavoids, inulin, glycosides, steroids, phlobatannins, terpenoids, phenols, and naphthoquinone. These phytochemicals have less toxicity and side effects [2–5]. The medicinal plant *Heliotropium europaeum* belongs to the Boraginaceae family, grown in summer, and is a heliotrope [6]. This toxic and medicinal plant is distributed in Middle Eastern countries, for instance, Saudi Arabia, Iraq, Syria, Iran, and Egypt, and Mediterranean countries, for instance, Turkey, Spain, Greece, France, Bosnia, Italy, Albania, Monaco, and Croatia, and is introduced accidently in Australia [7, 8]. *Heliotropium europaeum* has a poisonous and therapeutic effect, comprises pyrrolizidine which is an important alkaloid, and shows therapeutic properties such as antitumor, insecticidal, hepatotoxic, antibacterial, antifungal, mutagenic, teratogenic, mydriatic, and antispasmodic [9–11]. In this research study, the anti-inflammatory, anticancer (MCF-7, 3T3, and HeLa cell lines), and brine shrimp lethality assay and FTIR studies of the extract and fractions of the whole plant of *Heliotropium europaeum* are examined.

## 2. Materials and Methods

### 2.1. Plant Materials

The plant material (whole plant) of *Heliotropium europaeum* was collected from Dera Bugti, Balochistan, Pakistan, and identified by Prof. Dr. Rasool Bakhsh Tareen and Dr. Shazia Saeed, Department of Botany, University of Balochistan, Quetta, Pakistan, and was deposited in the Herbarium, Department of Botany, University of Balochistan, Quetta, Pakistan, with voucher number QUETTA000016.

### 2.2. Extraction of the Plant Material (Whole Plant) of *Heliotropium europaeum*

The whole plant of *Heliotropium europaeum* was washed with tap water and then rinsed with distilled water in order to reduce contamination which occurred during transportation and handling and shade dried for one month. This is due to the radiation from sunlight that destroys bioactive compounds present in the whole plant of *Heliotropium europaeum*. The dried whole plant was grinded in a mechanical grinder, and then, 12 kg powdered plant material was soaked in 20 litres of methanol, kept for 7 days, and shaken daily. After a 7-day period, the methanol-containing whole plant of *Heliotropium europaeum* was filtered with Whatman filter paper No. 1 and concentrated under reduced pressure at temperature below 55°C in a rotary evaporator. The dried semisolid whole-plant methanolic extract of *Heliotropium europaeum* (WMEHE) was 288 g. This crude extract 10 g was examined for biological activities such as anti-inflammatory, brine shrimp lethality assay, and anticancer MCF-7 cell line, anticancer 3T3 cell line, and anticancer HeLa cell line activities and FTIR analysis while the remaining extract was fractionated with solvents, for instance, n-hexane, aqueous solution, butanol, ether, dichloromethane, chloroform, and tetrachloromethane [12, 13].

### 2.3. Fractionation of the Crude Extract

The crude extract was fractionated with solvents such as n-hexane, aqueous solution, butanol, ether, dichloromethane, chloroform, and tetrachloromethane to form the whole-plant n-hexane fraction of *Heliotropium europaeum* (WHFHE) 3 g, whole-plant aqueous fraction of *Heliotropium europaeum* (WAFRR) 121.8 g, whole-plant butanol fraction of *Heliotropium europaeum* (WBFHE) 26 g, whole-plant ether fraction of *Heliotropium europaeum* (WEFHE) 0.2 g, whole-plant dichloromethane fraction of *Heliotropium europaeum* (WDFHE) 0.1 g, whole-plant chloroform fraction of *Heliotropium europaeum* (WCFHE) 0.3 g, and whole-plant tetrachloromethane fraction of *Heliotropium europaeum* (WTFHE) 0.1 g. The whole-plant n-hexane fraction of *Heliotropium europaeum* (WHFHE), whole-plant aqueous fraction of *Heliotropium europaeum* (WAFRR), and whole-plant butanol fraction of *Heliotropium europaeum* (WBFHE) were examined for anticancer, anti-inflammatory, and brine shrimp lethality assay and FTIT analysis while the whole-plant ether fraction of *Heliotropium europaeum* (WEFHE), whole-plant dichloromethane fraction of *Heliotropium europaeum* (WDFHE), whole-plant chloroform fraction of *Heliotropium europaeum* (WCFHE), and whole-plant tetrachloromethane fraction of *Heliotropium europaeum* (WTFHE) were examined for FTIR analysis [12, 13].

### 2.4. Anti-Inflammatory Assay

For the anti-inflammatory assay, the oxidative burst assay using the chemiluminescence technique was used.

### 2.5. Oxidative Burst Assay Using the Chemiluminescence Technique

In this technique, 25 *μ*l diluted whole blood in HBSS^++^ containing magnesium chloride and calcium chloride (Sigma, St. Louis, USA) and 25 *μ*l of the extract and fractions of medicinal plants were incubated for 15 min at 37°C in the thermostat chamber of a luminometer (Labsystems, Helsinki, Finland) and then plated in 96-well plates (Costar, NY, USA). Control wells contain HBSS^++^ and cells while blank wells contain HBSS^++^. 25 *μ*l luminol (Sigma Chemical Co., St. Louis, MO, USA) and 25 *μ*l serum opsonized zymosan (Sigma Chemical Co., St. Louis, MO, USA) were added into each well. In terms of relative light units, the level of ROS was recorded in a luminometer. In this assay, ibuprofen with IC_50_11.2 ± 1.9 is used as a standard drug [14].

### 2.6. MTT Assay (MCF-7 Cell Lines, 3T3 Cell Lines, and HeLa Cell Lines)

The MCF cell line, 3T3 cell line, and HeLa cell line were purchased from the American Type Culture Collection (ATCC). In this assay, Dulbecco's modified Eagle's medium containing ten percent fetal bovine serum and two percent antibiotics such as streptomycin with 100 *μ*g/ml and penicillin with 100 IU/ml was used for culturing MCF-7 cell lines, 3T3 cell lines, and HeLa cell lines which were then kept in five percent CO_2_ and incubated at 37°C. MCF-7 cells, 3T3 cells, and HeLa cells were harvested when confluency was developed and 5 × 10^4^ cells per well were plated in a 96-well flat. After 24 hours, the extract and fractions of medicinal plants with 50 *μ*g/ml were added and then incubated for 48 hours. After incubation, the extract/fractions were removed. To each well, 100 *μ*l with concentration of 0.5 mg/ml MTT was added and kept in an incubator for 4 hours at 37°C. MTT was reduced into formazan crystals which were then dissolved in 100 *μ*l DMSO and was taken at 570 nm absorbance using a microplate reader (SpectraMax Plus, Molecular Devices, CA, USA). In this assay, doxorubicin was used as a standard drug for the MCF-7 cell line and HeLa cell line while cycloheximide was used as a standard drug for the 3T3 cell line. The decrease in viable cells or percent inhibition was calculated with the help of the following formula:

(1) $$\begin{array}{c} \%\text{inhibition}=100-\left(\frac{\text{mean of O}.\text{D}.\text{of test compound}-\text{mean of O}.\text{D}.\text{of negative control}}{\text{mean of O}.\text{D}.\text{of positive control}-\text{mean of O}.\text{D}.\text{of negative control}}\times 100\right). \end{array}$$

For the calculation of IC_50_ 20 mM stock solution of the extract/fractions, diluted into working solution with 50 *μ*M, and then in order to get less than 50 percent inhibition, working solution is further diluted in serial dilutions. With the help of EZ-Fit5 software, IC_50_ is calculated [15].

### 2.7. Brine Shrimp Lethality Assay

B-Hatching techniques were used for the evaluation of toxicity.

### 2.8. B-Hatching Techniques

In this B-Hatching technique, scatter 50 mg of brine shrimp eggs in a hatching tray which was already half filled with filtered brine solution. Put it in an incubator at 37°C for 2 days. Take 20 mg of the extract and fractions of medicinal plants, and dissolve it in 2 ml of solvent such as methanol. Transfer 5, 50, and 500 *μ*l from this solution to 3 vials, and bring the concentration to 10, 100, and 1000 *μ*g/ml. Overnight, allow the solvent to evaporate. With the help of a Pasteur pipette, put 30 larvae per vial. Add 5 ml seawater. Under illumination, for 24 hours, incubate it at 25-27°C. For positive and negative controls, add a reference cytotoxic drug along with solvent in other vials. Etoposide was the standard drug used in this research study with 7.4625 *μ*g/ml. For the determination of LD_50_, the Finney computer program was used [16].

### 2.9. FTIR Analysis

The extract and fractions of the plant were dried for the analysis of FTIR. In FTIR analysis, the extract and fractions of the plant with the concentration of 10 mg were encapsulated in the pellet of 100 mg of KBr, for the preparation of the disc of the translucent sample which was then loaded in the FTIR spectroscope (Shimadzu, IRAffinity-1, Japan) [17].

## 3. Results

One extract and seven fractions were extracted and fractionated, respectively, from the whole plant of *Heliotropium europaeum*. Plant material (gm), yield (gm), and percentage yield of the extract and fractions of the whole plant of *Heliotropium europaeum* are shown in Table 1.

1. Whole-plant methanolic extract of *Heliotropium europaeum* (WMEHE)
2. Whole-plant n-hexane fraction of *Heliotropium europaeum* (WHFHE)
3. Whole-plant aqueous fraction of *Heliotropium europaeum* (WAFHE)
4. Whole-plant butanol fraction of *Heliotropium europaeum* (WBFHE)
5. Whole-plant ether fraction of *Heliotropium europaeum* (WEFHE)
6. Whole-plant dichloromethane fraction of *Heliotropium europaeum* (WDFHE)
7. Whole-plant chloroform fraction of *Heliotropium europaeum* (WCFHE)
8. Whole-plant tetrachloromethane fraction of *Heliotropium europaeum* (WTFHE)

The whole-plant butanol fraction of *Heliotropium europaeum* (WBFHE) showed anti-inflammatory activity on ROS having IC_50_14.7 ± 2.5 while the extract and other fractions of the whole plant of *Heliotropium europaeum* exhibited no anti-inflammatory activity. The anti-inflammatory activity of the extract and fractions of the whole plant of *Heliotropium europaeum* is shown in Table 2.

**Figure 1:**
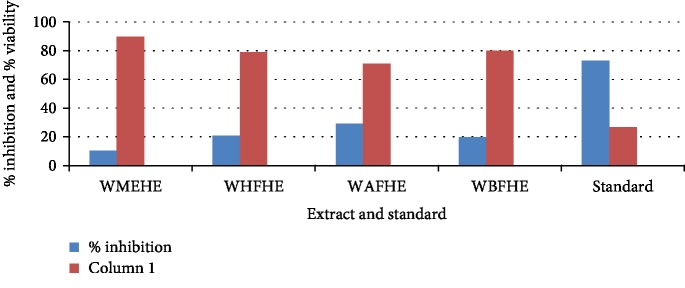
Anticancer assay (MCF-7) of the extract and fractions of the whole plant of *Heliotropium europaeum*.

**Figure 2:**
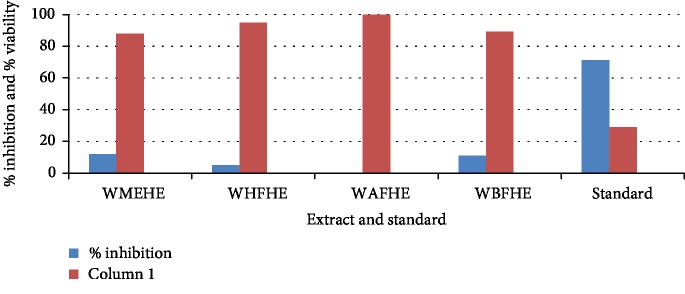
Anticancer assay (3T3) of the extract and fractions of the whole plant of *Heliotropium europaeum*.

**Figure 3:**
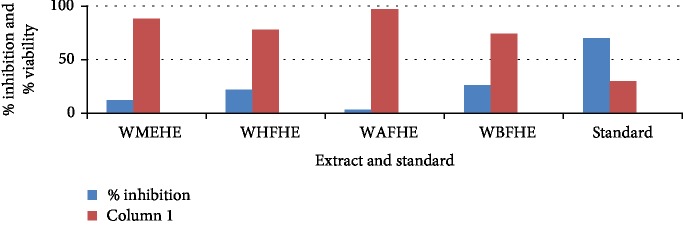
Anticancer assay (HeLa cell line) of the extract and fractions of the whole plant of *Heliotropium europaeum*.

**Figure 4:**
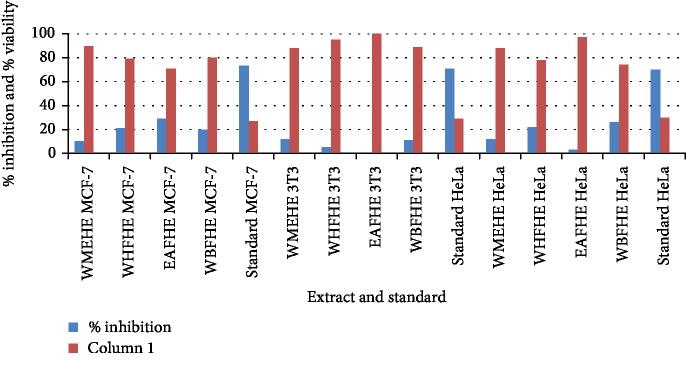
Anticancer assay (MCF-7, 3T3, and Hela cell lines) of the extract and fractions of the whole plant of *Heliotropium europaeum*.

None of the extract and fractions of the whole plant of *Heliotropium europaeum* exhibited anticancer (MCF-7, 3T3, and HeLa cell lines) activities. Anticancer activities of the extract and fractions of the whole plant of *Heliotropium europaeum* are shown in Tables 3–5 and Figures 1–4.

The whole-plant aqueous fraction of *Heliotropium europaeum* (WAFHE) and whole-plant butanol fraction of *Heliotropium europaeum* (WBFHE) showed lethality at high concentration while at low concentration, no toxicity was shown. The whole-plant methanolic extract of *Heliotropium europaeum* (WMEHE) and whole-plant n-hexane fraction of *Heliotropium europaeum* (WHFHE) exhibited no toxicity. The results of the brine shrimp lethality assay of the extract and fractions of the whole plant of *Heliotropium europaeum* are shown in Table 6.

**Figure 5:**
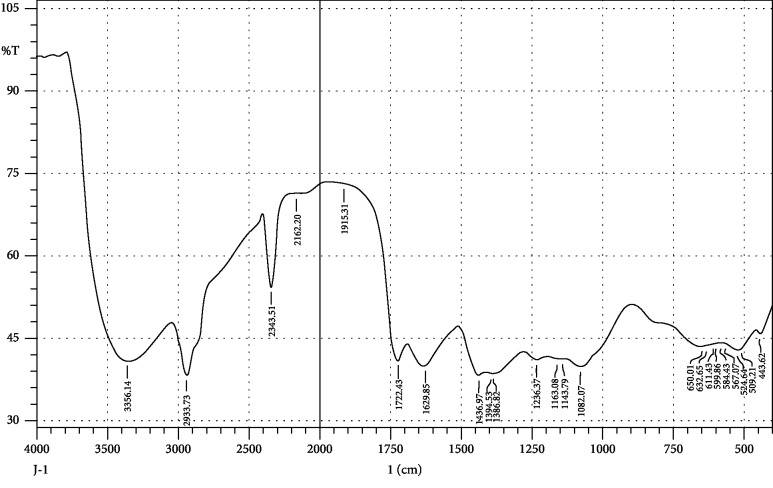
FTIR spectrum analysis of the whole-plant methanol extract of *Heliotropium europaeum* (WMEHE).

**Figure 6:**
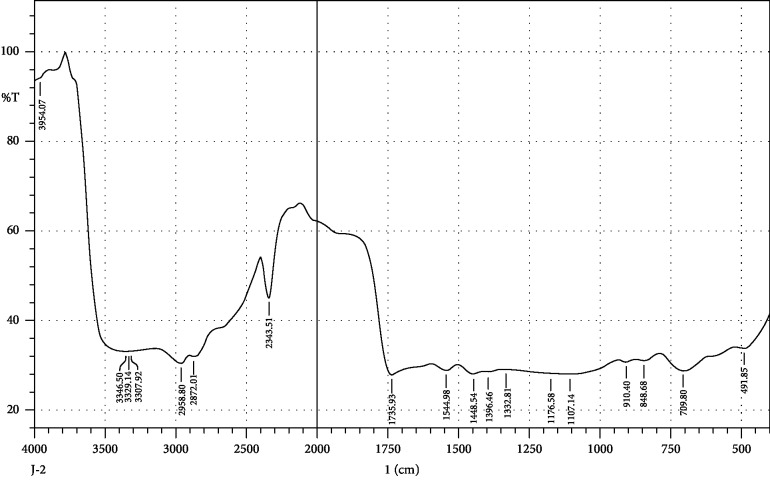
FTIR spectrum analysis of the whole-plant hexane fraction of *Heliotropium europaeum* (WHFHE).

**Figure 7:**
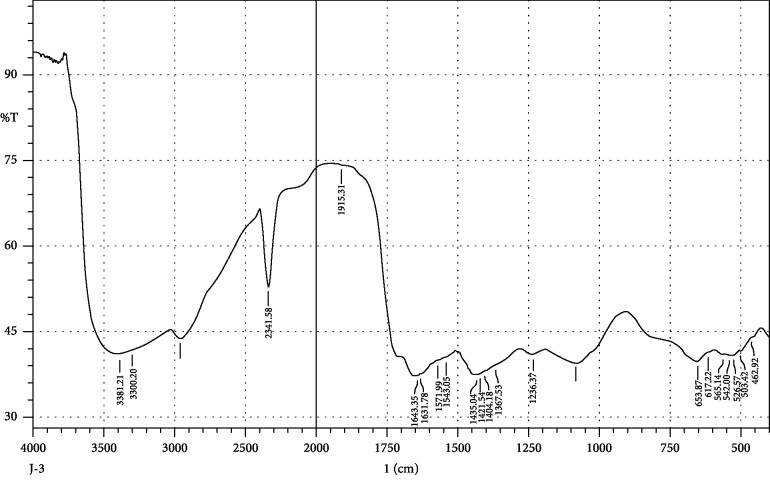
FTIR spectrum analysis of the whole-plant aqueous fraction of *Heliotropium europaeum* (WAFHE).

**Figure 8:**
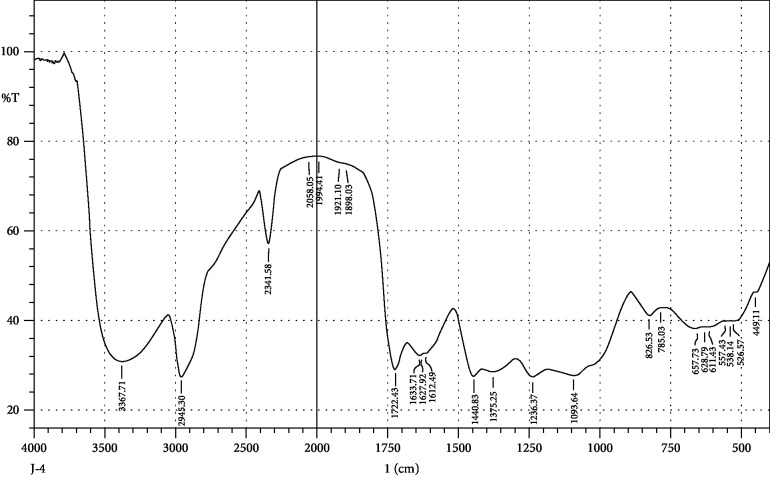
FTIR spectrum analysis of the whole-plant butanol fraction of *Heliotropium europaeum* (WBFHE).

**Figure 9:**
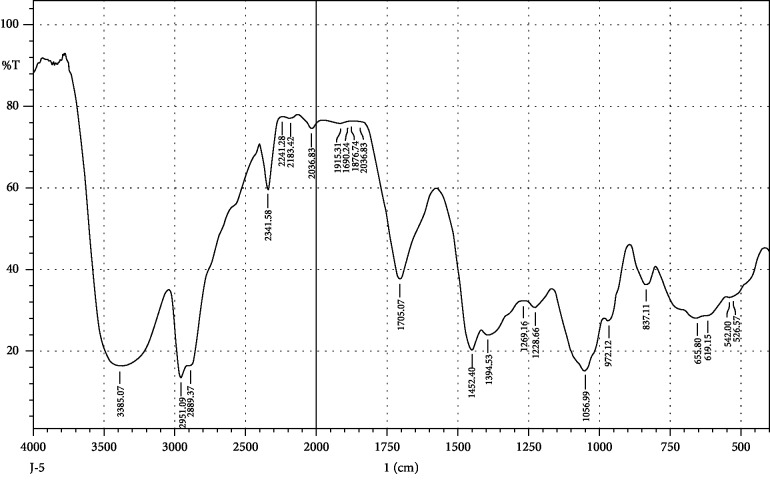
FTIR spectrum analysis of the whole-plant ether fraction of *Heliotropium europaeum* (WEFHE).

**Figure 10:**
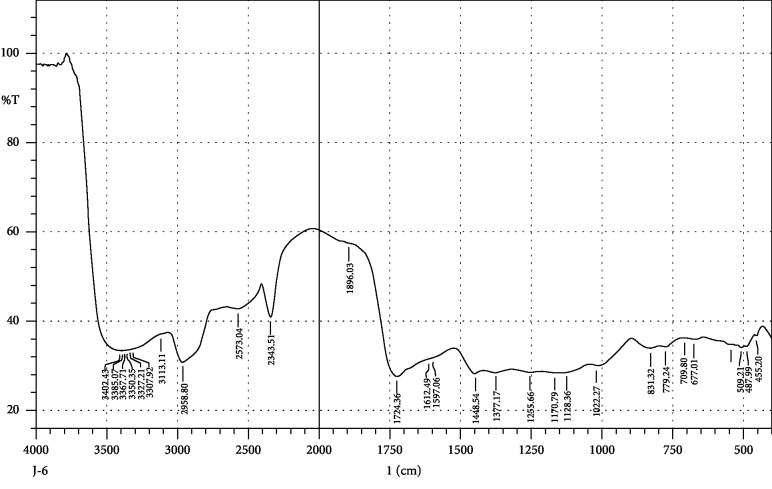
FTIR spectrum analysis of the whole-plant dichloromethane fraction of *Heliotropium europaeum* (WDFHE).

**Figure 11:**
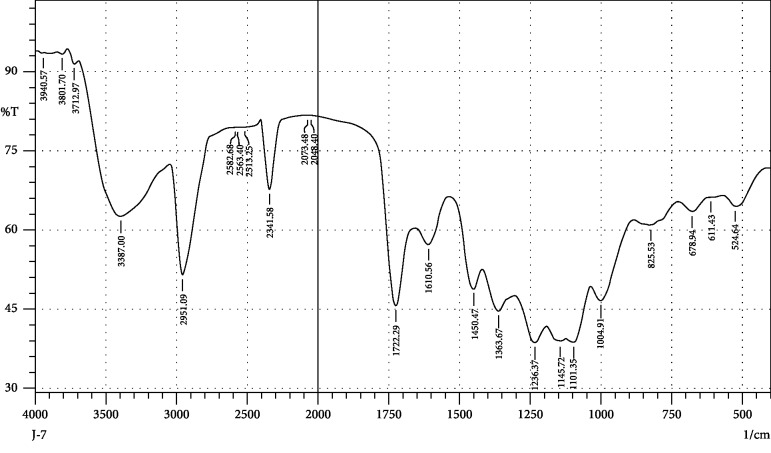
FTIR spectrum analysis of the whole-plant chloroform fraction of *Heliotropium europaeum* (WCFHE).

**Figure 12:**
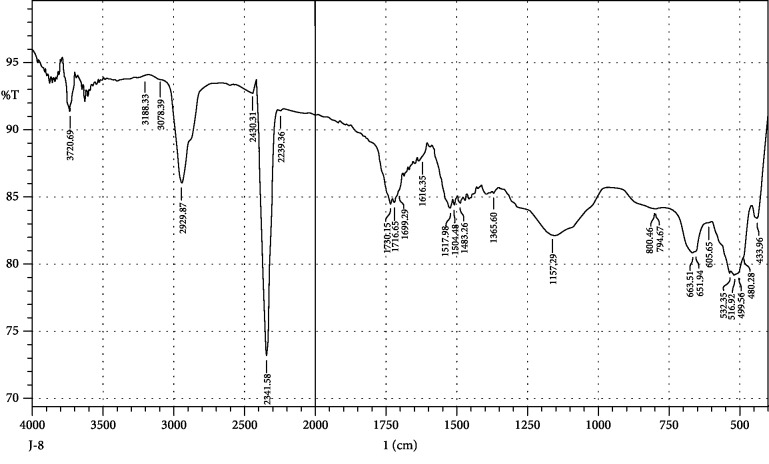
FTIR spectrum analysis of the whole-plant tetrachloromethane fraction of *Heliotropium europaeum* (WTFHE).

FTIR interpretation of the extract and fractions of *Heliotropium europaeum* showed the functional groups for the aromatic compounds, phenols, carboxylic acids, esters, alkanes, alkenes, alcohols, alkyl halides, sulfate esters, phosphines, silanes, nitriles, thiols, amines, phosphoric acids, and nitro compounds. FTIR analysis of the extract and fractions of the whole plant of *Heliotropium europaeum* is shown in Tables 7–14. FTIR analysis of the extract and fractions of the whole plant of *Heliotropium europaeum* is shown in Figures 5–12.

## 4. Conclusion

In this research study, the whole-plant butanol fraction of *Heliotropium europaeum* (WBFHE) showed anti-inflammatory activity on ROS having IC_50_14.7 ± 2.5 while the extract and other fractions of the whole plant of *Heliotropium europaeum* exhibited no anti-inflammatory activity. None of the extract and fractions of the whole plant of *Heliotropium europaeum* exhibited anticancer (MCF-7, 3T3, and HeLa cell lines) activities. The whole-plant aqueous fraction of *Heliotropium europaeum* (WAFHE) and whole-plant butanol fraction of *Heliotropium europaeum* (WBFHE) showed lethality at high concentration while at low concentration, no toxicity was shown. The whole-plant methanolic extract of *Heliotropium europaeum* (WMEHE) and whole-plant n-hexane fraction of *Heliotropium europaeum* (WHFHE) exhibited no toxicity. FTIR interpretation showed the functional groups for the aromatic compounds, phenols, carboxylic acids, esters, alkanes, alkenes, alcohols, alkyl halides, sulfate esters, phosphines, silanes, nitriles, thiols, amines, phosphoric acids, and nitro compounds.

## Figures and Tables

**Table 1:** Plant material (gm), yield (gm), and percentage yield of the extract and fractions of the whole plant of *Heliotropium europaeum*.

Plant	Extract and fractions	Plant material	Plant material (gm)	Yield (gm)	Percentage yield %Yield = (W1/W2) × 100 W1 = yield (gm) W2 = plant material (gm)
*Heliotropium europaeum*	WMEHE	Whole plant	12000	288	2.4%
	WHFHE	Whole plant	12000	3	0.025%
	WAFHE	Whole plant	12000	121.8	1%
	WBFHE	Whole plant	12000	26	0.2%
	WEFHE	Whole plant	12000	0.2	0.0016%
	WDFHE	Whole plant	12000	0.1	0.0008%
	WCFHE	Whole plant	12000	0.3	0.0025%
	WTFHE	Whole plant	12000	0.1	0.0008%

**Table 2:** Anti-inflammatory activities of the extract and fractions of the whole plant of *Heliotropium europaeum*.

S. no.	Extract/fraction/Std. drug	Conc. (*μ*g/ml)	% inhibition/stimulation	IC_50_ ± S.D.
1	WMEHE	25	33.2	—
2	WHFHE	250, 50, 10	—	>250
3	WAFHE	250, 50, 10	—	>250
4	WBFHE	250, 50, 10	—	14.7 ± 2.5
5	Ibuprofen		73.2	11.2 ± 1.9 *μ*g/ml

**Table 3:** Anticancer assay (MCF-7) of the extract and fractions of the whole plant of *Heliotropium europaeum*.

S. no.	Extract/fraction/Std. drug	Conc. (*μ*g/ml)	% inhibition/stimulation	% viability	IC_50_ ± S.D.
1	WMEHE	50	10.38	89.62	Inactive
2	WHFHE	50	20.89	79.11	Inactive
3	WAFHE	50	29.19	70.81	Inactive
4	WBFHE		19.96	80.04	Inactive
5	Doxorubicin	50	73.23	26.77	0.80 ± 0.05

**Table 4:** Anticancer assay (3T3) of the extract and fractions of the whole plant of *Heliotropium europaeum*.

S. no.	Extract/fraction/Std. drug	Conc. (*μ*g/ml)	% inhibition/stimulation	% viability	IC_50_ ± S.D.
1	WMEHE	30	12	88	Inactive
2	WHFHE	30	5	95	Inactive
3	WAFHE	30	0	100	Inactive
4	WBFHE		11	89	Inactive
5	Cycloheximide	30	71	29	0.85 ± 0.07

**Table 5:** Anticancer assay (HeLa cell line) of the extract and fractions of the whole plant of *Heliotropium europaeum*.

S. no.	Extract/fraction/Std. drug	Conc. (*μ*g/ml)	% inhibition/stimulation	% viability	IC_50_ ± S.D.
1	WMEHE	30	12	88	Inactive
2	WHFHE	30	22	78	Inactive
3	WAFHE	30	3	97	Inactive
4	WBFHE	30	26	74	Inactive
5	Doxorubicin	30	70	30	1.2 ± 0.4

**Table 6:** Brine shrimp lethality bioassay of the extract and fractions of the whole plant of *Heliotropium europaeum*.

S. no	Extract and fractions	Dose (*μ*g/ml)	No. of shrimps	No. of survivors	% mortality	LD50 (*μ*g/ml)	Std. drug	LD_50_ (*μ*g/ml)
1	WMEHE	10	30	30	0%		Etoposide	7.4625
		100	30	30	0%			
		1000	30	30	0%			

2	WHFHE	10	30	30	0%		Etoposide	7.4625
		100	30	30	0%			
		1000	30	30	0%			

3	WAFHE	10	30	30	0%		Etoposide	7.4625
		100	30	30	0%			
		1000	30	28	6.66%			

4	WBFHE	10	30	30	0%		Etoposide	7.4625
		100	30	29	3.33%			
		1000	30	19	36.66%			

**Table 7:** FTIR analysis of the whole-plant methanol extract of *Heliotropium europaeum* (WMEHE).

S. no.	Peak	Height	Corr. height	Base (*H*)	Base (*L*)	Area	Corr. area	Interpretation
1	509.21	56.905	0.147	511.14	457.13	18.872	0.075	S-S disulfide
2	567.07	56.1	0.103	576.72	563.21	4.82	0.008	C-Br
3	1082.07	60.349	3.539	1132.21	891.11	85.528	0.029	C-O stretch of esters
4	1143.79	58.889	0.029	1155.36	1134.14	8.188	0.472	C-O stretch of ethers
5	1163.08	58.869	0.101	1195.87	1157.29	14.799	0.339	C-H wag (-CH2X)
6	1236.37	59.02	1.042	1280.73	1197.79	31.687	0.021	P-H bending of phosphine
7	1394.53	61.578	0.131	1408.04	1388.75	7.997	0.192	S=O sulfate esters
8	1436.97	61792	1.586	1510.26	1425.4	32.271	0.035	Ar C-C stretch
9	1629.85	60.249	0.435	1635.64	1512.19	44.756	0.005	Ar CH=CHR aromatic alkenes
10	1722.43	59.069	0.196	1724.36	1691.57	12.183	0.569	C=O stretch of esters
11	2162.2	28.864	0.359	2193.06	1977.04	30.999	17.363	Si-H silane
12	2343.51	45.883	14.637	2405.23	2208.49	37.791	90.882	P-H phosphine sharp
13	2933.73	61.877	13.199	3045.6	2407.16	173.618		C-H stretch of alkanes
14	3356.14	59.289	27.719	3786.27	3047.53	214.072		ArO-H H-bonded of phenols

**Table 8:** FTIR analysis of the whole-plant hexane fraction of *Heliotropium europaeum* (WHFHE).

S. no.	Peak	Height	Corr. height	Base (*H*)	Base (*L*)	Area	Corr. area	Interpretation
1	709.8	71.892	4.287	794.67	514.99	144.151	8.191	C-H out of plane of aromatics
2	848.68	69.698	0.827	875.68	796.6	40.345	0.504	C-H out of plane of aromatics
3	910.4	69.82	0.326	939.33	877.61	31.969	0.156	N-H wag amines
4	1107.14	72.618	0.505	1136.07	941.26	106.771	1.709	C-N stretch of amines
5	1176.58	72.667	0.338	1327.03	1138	105.055	0.476	C-O stretch of esters
6	1332.81	71.496	0.004	1340.53	1328.95	6.308	0	N-O sym stretch of nitro compound
7	1396.46	72.004	0.174	1415.75	1342.46	40.297	0.101	S=O sulfate esters
8	1448.54	72.57	1.23	1502.55	1417.68	46.721	0.915	Ar C-C stretch
9	1544.98	71.676	1.37	1595.13	1404.48	48.746	0.937	N-O asym stretch of nitro compound
10	1735.93	72.649	15.406	1907.6	1597.06	131.605	17.518	C=O stretch of esters
11	2872.01	68.436	1.523	2899.01	2401.38	200.732	10.737	C-H stretch of alkanes
12	2958.8	69.969	2.214	3138.18	2900.94	118.397	2.981	C-H stretch of alkanes
13	3307.92	67.218	0.013	3311.78	3140.11	82.598	0.112	ArO-H H-bonded of phenols
14	3329.14	67.239	0.01	3336.85	3311.78	12.149	0.001	N-H stretch of amines

**Table 9:** FTIR analysis of the whole-plant aqueous fraction of *Heliotropium europaeum* (WAFHE).

S. no.	Peak	Height	Corr. height	Base (*H*)	Base (*L*)	Area	Corr. area	Interpretation
1	503.42	58.347	0.299	507.28	464.84	15.692	0.115	S-S disulfide
2	542	59.12	0.12	553.57	538.14	5.98	0.012	C-Br stretch
3	1085.92	60.482	4.956	1193.94	914.26	104.059	7.441	C-N stretch of amines
4	1236.37	58.967	0.869	1278.81	1195.87	31.726	0.395	C-H wag (-CH2X)
5	1367.53	60.857	0.077	1369.46	1290.38	31.112	0.116	S=O esters
6	1404.18	61.898	0.071	1406.11	1369.40	15.154	0.028	S=O sulfate esters
7	1435.04	62.618	0.128	1438.9	1425.4	5.75	0.012	Ar C-C stretch
8	1543.05	59.517	0.168	1546.91	1510.26	14.182	0.059	N-O asym stretch of nitro compounds
9	1571.99	60.098	0.077	1573.91	1558.48	6.125	0.008	N=O nitroso
10	1631.78	62.413	0.105	1633.71	1575.84	23.792	0.041	NH2 in plane bend of amine
11	1643.35	62.75	0.205	1649.14	1635.64	5.774	0.021	C=N
12	2954.95	56.255	3.937	3026.31	2403.3	169.441	7.483	C-H stretch of alkanes
13	3300.2	58.334	0.054	3304.06	3028.24	100.312	0.428	ArO-H H-bonded of phenols
14	3381.21	58.978	0.035	3385.07	3305.99	30.353	0.027	N-H stretch of amines

**Table 10:** FTIR analysis of the whole-plant butanol fraction of *Heliotropium europaeum* (WBFHE).

S. no.	Peak	Height	Corr. height	Base (*H*)	Base (*L*)	Area	Corr. area	Interpretation
1	526.57	60.462	0.202	528.5	455.2	27.591	0.578	S-S disulfide
2	557.43	60.525	0.205	565.14	549.71	6.213	0.02	C-Br
3	787.03	57.485	0.066	792.74	771.53	7.873	0.008	C-Cl
4	1093.64	72.754	7.211	1190.08	896.9	146.453	17.993	C-O stretch of ethers
5	1236.37	72.97	2.73	1296.16	1192.01	56.711	2.085	C-H wag (-CH2X)
6	1375.25	72.012	1.477	1409.96	1298.09	59.987	1.356	CH2 and CH3 of alkanes
7	1440.83	72.733	4.684	1516.05	1417.86	48.245	3.253	S=O sulfate esters
8	1627.92	68.05	0.069	1629.85	1614.42	7.612	0.012	C=N
9	1722.43	71.371	15.773	1840.09	1681.93	54.691	8.927	Monomer C=O of carboxylic acids
10	2058.05	23.659	0.013	2059.98	2011.76	5.615	0.008	N=C in R-N=C=S
11	2945.3	72.963	18.244	3041.74	2407.16	207.79	34.031	C-H stretch of alkanes
12	3367.71	69.56	36.829	3693.68	3043.67	260.725	125.447	Dimer O-H of carboxylic acids

**Table 11:** FTIR analysis of the WEHE5 whole-plant ether fraction of *Heliotropium europaeum* (WEFHE).

S. no.	Peak	Height	Corr. height	Base (*H*)	Base (*L*)	Area	Corr. area	Interpretation
1	526.57	67.089	0.244	528.5	414.7	47.877	0.959	S-S disulfide
2	542	67.422	0.24	545.85	530.42	7.488	0.033	C-Br
3	837.11	64.225	6.395	894.97	804.32	37.173	3.73	C-Cl
4	1056.99	85.296	15.749	1168.86	987.55	121.948	29.855	P-H bending of phosphine
5	1228.66	69.684	2.584	1259.52	1170.79	43.885	1.595	C-H wag (-CH2X)
6	1269.16	68.233	0.066	1280.73	1261.451282.66	9.597	0.009	C-O stretch of carboxylic acids
7	1394.53	76.531	2.519	1417.68	1282.66	77.545	3.229	S=O sulfate esters
8	1452.4	80.038	12.112	1575.84	1419.61	70.087	7.949	CH2 and CH3 of alkanes
9	1705.07	62.731	30.359	1832.38	1577.77	71.348	27.206	Dimer C=O of carboxylic acids
10	2036.83	25.82	2.664	2129.41	1982.82	17.65	1.04	N=C in R-N=C=S
11	2183.42	23.243	0.678	2216.21	2131.34	9.575	0.187	Si-H silane
12	2341.58	40.615	13.598	2399.45	2249	25.344	5.458	P-H phosphine sharp
13	2889.37	83.769	1.082	2899.01	2401.38	176.297	0.617	C-H stretch of alkanes
14	2951.09	86.663	9.854	3035.96	2900.94	95.107	11.272	C-H stretch of alkanes
15	3385.07	83.763	45.421	3728.4	3037.89	387.188	210.043	Dimer OH of carboxylic acids

**Table 12:** FTIR analysis of the whole-plant dichloromethane fraction of *Heliotropium europaeum* (WDFHE).

S. no.	Peak	Height	Corr. height	Base (*H*)	Base (*L*)	Area	Corr. area	Interpretation
3	509.21	66.099	0.243	518.85	501.49	8.128	0.026	S-S disulfide
4	545.85	65.557	0.072	551.64	540.07	5.353	0.006	C-Br
5	677.01	64.418	0.489	700.16	644.22	24.94	0.179	=CH out of plane of alkene
7	779.24	66.065	0.653	796.6	717.52	36.174	0.265	N-H wag amines
8	831.32	66.341	1.118	896.9	798.53	45.718	0.919	C-H out of plane of aromatics
9	1022.27	70.209	1.238	1045.42	898.83	72.487	1.804	P-H bending of phosphine
10	1128.36	71.605	0.119	1134.14	1047.35	46.475	0.176	C-N stretch of amines
11	1170.79	71.839	0.309	1211.3	1136.07	41.235	0.197	C-O stretch of esters
12	1255.66	71.674	0.417	1307.74	1213.23	51.476	0.325	C-H wag (-CH2X)
13	1377.17	71.741	0.552	1411.89	1309.67	55.549	0.369	CH2 and CH3 of alkanes
14	1448.54	71.855	2.008	1521.84	1421.54	52.27	1.535	Ar C-C stretch of phenols
15	1597.06	86.317	0.075	1598.99	1523.76	36.514	0.088	NH2 in plane bend
16	1612.49	68.717	0.066	1614.42	1598.99	7.742	0.006	C=C stretch of alkenes
17	1724.36	72.514	14.099	1890.24	1616.35	117.083	16.523	C=O stretch of esters
19	2343.51	59.216	9.459	2405.23	2038.76	99.242	5.54	P-H phosphine sharp
20	2573.04	75.36	2.07	2654.05	2407.16	88.15	3.802	S-H sharp of thiols
21	2958.8	69.273	8.105	3061.03	2655.98	177.621	17.639	C-H stretch of alkanes
22	3113.11	63.003	0.016	3115.04	3062.96	22.368	0.011	=C-H stretch of alkenes
23	3307.92	66.138	0.071	331.78	3115.04	89.183	0.536	ArO-H H-bonded of phenols
24	3327.21	66.29	0.02	3329.14	3313.71	7.269	0.001	N-H stretch of amines
28	3402.43	66.579	0.045	3415.93	3394.72	10.093	0.008	N-H stretch of amines

**Table 13:** FTIR analysis of the whole-plant chloroform fraction of *Heliotropium europaeum* (WCFHE).

S. no.	Peak	Height	Corr. height	Base (*H*)	Base (*L*)	Area	Corr. area	Interpretation
1	524.64	35.914	3.296	565.14	405.05	27.586	1.549	S-S disulfide
2	611.43	34.252	0.048	617.22	567.07	9.095	0.017	C-Br
3	825.53	39.54	0.956	846.75	727.16	24.686	0.538	N-H wag amines
4	1004.91	53.907	5.508	1037.7	887.26	42.198	3.138	P-H bending of phosphine
5	1101.35	61.8	3.37	1124.5	1039.63	32.25	1.729	C-N stretch of amines
6	1145.72	61.54	1.113	1192.01	1126.43	26.692	0.607	C-O stretch of esters
7	1236.37	61.804	5.301	1305.81	1193.94	42.658	2.845	C-H wag (-CH2X)
8	1363.67	55.831	5.527	1417.68	1307.74	36.288	2.751	C-H rock of alkanes
9	1450.47	51.6	7.497	1535.34	1419.61	29.295	2.774	CH_2_ and CH_3_ of alkanes
10	1610.56	43.11	5.439	1656.85	1537.27	26.19	2.202	NH_2_ in plane bend of amines
11	1722.29	54.706	18.628	2029.11	1658.78	56.075	7.445	C=O stretch of esters
12	2073.48	18.552	0.007	2079.26	2054.19	2.234	0.001	N=C in R-N=C=S
13	2341.58	32.536	13.367	2405.23	2102.41	32.959	5.348	P-H phosphine sharp
14	2563.4	20.744	0.021	2571.11	2515.18	5.636	0.006	S-H sharp of thiols
15	2582.68	20.744	0.025	2611.62	2573.04	3.89	0.003	(O=)PO-H phosphoric acids
16	2951.09	48.718	22.524	3039.81	2613.55	71.029	20.094	C-H stretch of alkanes
17	3387	37.471	20.375	3685.97	3041.74	99.785	42.816	N-H stretch of amines

**Table 14:** FTIR analysis of the whole-plant tetrachloromethane fraction of *Heliotropium europaeum* (WTFHE).

S. no.	Peak	Height	Corr. height	Base (*H*)	Base (*L*)	Area	Corr. area	Interpretation
1	516.92	21.117	0.151	526.57	513.07	1.384	0.006	S-S disulfide
2	794.67	16.108	0.01	796.6	781.17	1.175	0.001	C-H out of plane of aromatics
3	800.46	16.113	0.045	958.62	796.6	11.715	0.077	C-H out of plane of aromatics
4	1157.29	18.074	2.67	1271.09	960.55	24.623	2.378	C-O stretch of esters
5	1365.6	14.92	0.202	1369.46	1350.17	1.335	0.011	S=O esters
6	1483.26	15.627	0.503	1490.97	1473.62	1.263	0.026	Ar C-C stretch of aromatics
7	1517.98	16.033	1.015	1548.84	1510.26	2.791	0.148	N-O asym stretch of nitro compounds
8	1616.35	12.206	0.149	1618.28	1598.99	1.046	0.016	C=N
9	1716.65	15.575	0.436	1722.43	1703.14	1.383	0.024	C=O of carboxylic acids
10	1730.15	15.697	0.748	1799.59	1724.36	4.615	0.069	C=O stretch of esters
11	2239.36	8.703	0.111	2249	2214.28	1.358	0.008	Si-H silane
12	2929.87	13.999	7.508	3053.32	2725.42	13.854	4.244	C-H stretch of alkanes
13	3078.39	6.297	0.044	3136.25	3072.6	1.761	0.01	Ar-H stretch of aromatics
14	3188.33	6.016	0.004	3190.26	3153.61	0.981	0	Dimer O-H of carboxylic acids

## Data Availability

The data used to support the findings of this study are available from the corresponding author upon request.
